# Continuity and Change

**Published:** 1925

**Authors:** T. Carwardine

**Affiliations:** Surgeon to the Bristol Royal Infirmary


// The Bristol

Medico-Chirurgical Journal

"Scire est nescire, nisi id me
Scire alius scierit."

WINTER, 1925.

### CONTINUITY AND CHANGE.

The Presidential Address, delivered on October 14th, 1925, at the opening of the Fifty-third Session of the Bristol Medico-Chirurgical Society

BY

T. CARWARDINE, M.S., F.R.C.S.,
*Surgeon to the Bristol Royal Infirmary.*

IF we wish to discern the origin of all natural things we must take a wide view and look to the Heavens. The impression to the observer is one of movement, change and order. The planets move in the same direction, in orbits having approximately similar planes, with their axes inclined to those planes at a like angle, and at distances which have a numerical relationship (Bode's Law). The visible stars, largely aggregated in a circumferential view as the "milky way," are a teeming multitude in various stages of evolution, self-luminous. They are suns, perhaps with non-luminous worlds rotating round them. The spectra of the stars comprise three groups: (1) Those in which hydrogen, helium and magnesium are prominent, hydrogen predominating in the brightest star Sirius; (2) those with strong metallic lines similar to the solar spectrum; and (3) those which suggest coolness and chemical combinations (hydrocarbons and metallic vapours), "the last stage of a sun—when it has cooled down." The nebulæ may be gaseous and irregular (Orion), or of appearance suggesting solidarity and orbital movement (Andromeda). The spectroscopic telescope has identified hydrogen in their spectra, and helium has been proved to be present in the nebula of Orion. It is significant that these two elements head the list in the atomic order.

Modern science teaches that the atom consists of a nucleus of positive electricity, circulating around which are negatively charged electrons which vary in number precisely with the position of the atom in the atomic scale. Hydrogen, the first, has one electron: the last on the list is uranium, with ninety-two electrons. The physical and chemical properties depend on the electrons. There are many possible orbits for the rotation of the electrons. That of hydrogen is most stable at the inner orbit; but the fine lines in the hydrogen spectrum reveal that there are other possible orbits; and when an electron passes from one orbit to another energy is given out which manifests itself as radiation. Helium has two electrons, circulating in different orbits; whilst lithium, the third on the list, has three electrons circulating in three orbits; and so on. The third electron of lithium has a very elliptical orbit, resembling that of a comet in the solar system. The orbits are always ready to fill up their complement of electrons. When the full number is incomplete the atom is less stable, when complete a very stable element is formed. For new elements to result from the addition of electrons there must be fresh groupings and the occupation of further orbits—at first unstable and later stable when the complement is full. A beautiful experiment is illustrated by Sir Wm. Bragg: magnets floating on water arrange themselves in a circle around an electro-magnet in the centre of a bath. When additional magnets are added they float inwards and form a second circle.

From the classification of atoms according to the number of their electrons some new elements have been, and some remain to be, discovered. From the arrangement of atoms according to their groupings, based upon the satisfaction of the orbits by electrons and their physical characters, the elements of one group may be shown to have features in common with those of other groups, and the relationship indicated by connecting the related elements together by lines. The writer has found that zinc is a specific in syphilis. Now testing the table by this and other known remedies for diseases caused by spirochætes, arsenic, bismuth and mercury, it will be observed that zinc and arsenic (30 and 33) are related to mercury and bismuth (80 and 83) in the next group.

A remarkable change has occurred in our ideas of mass, which is not the constant unit formerly taught. Mass increases with the velocity. This was asserted in the theory of relativity (Einstein), and experiments have confirmed it. Since orbits are elliptical, both in the solar system and in the atom, the mass of revolving bodies varies with their velocity in different parts of the orbits.

The discovery of X-rays and Radium has afforded much information in minute structure. X-rays are transverse waves like ordinary light, but 10,000 times smaller and far more penetrating. They are not electrified, and are not deflected in an electro-magnetic field. When they fall on a sheet of metal they liberate electrons from the metal. The X-ray spectra of the elements follow the atomic numbers (not weights), and have led to the discovery of new elements. They help us to see the atom indirectly. Radium tells us what the atom does. It gives off helium. The a-particles show the spectrum of helium. The helium atom from radium starts with a velocity of 10,000 miles a second, goes through the molecules it meets with, and completes its course in about three inches of air. By the induction of fog the track of the a-particles has been photographed ; and, in rare instances, the impact between atoms when there has been a direct hit ; although in most cases atoms go through one another.

When combinations of atoms produce molecules the mode of attachment tends to take on a definite form. Chemists have figured "ring" molecules and "chain" molecules for many years on theoretical grounds ; and X-ray spectra have proved them to be a real basis of structure, except that molecules must be regarded as having three dimensions, not two as illustrated in books on chemistry. Carbon may exist as diamond or graphite : in the former the carbon atoms (each with six electrons) have the hexagonal ring arrangement ; in the latter the chain. Diamond is very hard, whereas graphite is one of the best lubricants. X-rays have shown that graphite is a lubricant because the molecules are in chains or planes, with a wide separation between the layers. Soap molecules are described by Sir Wm. Bragg as standing on end with different atoms at the poles, the deeper poles having atoms which associate with other molecules. Thus molecules tend to cling in a definite way, and this is the foundation of crystalline form.

The photographic X-ray spectra of numerous substances have shown them to be of crystalline nature in internal design. Moreover, the construction of some crystals has been determined. Thus the quartz unit contains three molecules arranged in a screw-like form, dextro- or lævo-rotatory. In a few cases the X-ray spectrometer has decided the place of every atom in the unit of pattern. The atomic arrangement in metals, in gold leaf for instance, is in effect crystalline ; and the line of fracture is between the crystals. The hardening of metals is effected by introducing substances which jamb the sliding planes of the crystals.

Many facts indicate that matter may undergo change or exhibit a degree of activity. Phosphorus and carbon exist in two or more forms. Iron rails gradually turn into steel under stress. Within certain temperatures tin may become a powder—the " tin pest " of organ pipes. Alloys of non-magnetic elements may be magnetic. Gold will diffuse through lead when the two are pressed together for long. Rocks undergo metamorphosis. Metals exposed to sunlight, or when rubbed together in the dark, may affect a photographic plate even through an opaque film.

Thus far we have traced the element, the atom, the molecule, the crystal, and the substance.

### CONTINUITY AND CHANGE IN LIFE.

When what we call Life appears it is in association with a much more complex molecule—protoplasm ; and animal life is entirely dependent upon plant life. For the existence of plants there are three factors : sunlight, chlorophyll and the nitrifying bacteria. Diatoms, unicellular algæ containing chloroplasts, form the ultimate food of fish. In spring every square yard of the sea contains a million, and their flinty skeletons cover millions of square miles of the ocean bed.

There are several physiological processes in plants which resemble those in animals. They have no specialised lungs, but *respire* freely. They inspire through the stomata into air-spaces which expand under the influence of sunlight, and so take in $CO_2$ and O. They also absorb moisture through the roots and *transpire*, giving humidity to the atmosphere : an acre of wheat during its life-time gives off a thousand tons of water. Plants have a circulatory system comprising tracheids, vessels and sieve-tubes, through which the liquid nourishment passes, and the carbohydrates built up in the leaf are conveyed into the stem through the veins. Their assimilation is of an order unequalled in the animal kingdom, for they can make protoplasm from inorganic material out of simple molecules containing two elements ($H_2O$, $CO_2$, $NH_3$). They manufacture complex proteins and carbohydrates for animal life to feed upon: and animals subsist by breaking those substances down, returning the disintegrated molecules back to the vegetable kingdom for re-manufacture. Water is absorbed by the roots, carbon compounds (sugars) are formed in the leaves, and nitrogenous material supplied at the roots.

*Carbohydrates.* Under the action of subdued sunlight upon chlorophyll (photosynthesis) sugar and starch are formed, probably through the intermediate production of formaldehyde. The sugar is for immediate use during the day, and the excess is stored as starch for conversion into sugar during the night. The process is analogous to the glycogenic function in animals. *Chlorophyll,* upon which animal life ultimately depends, is of particular interest to medical men, and a wonderful substance with the following uses :—

1. Photosynthesis in leaves.
2. Stored in the liver of many invertebrates.
3. Resembles hæmoglobin and probably its source.
4. Replenishes the O supply of the world.
5. Indirectly (through Hb) acts as the O carrier in higher animals.
6. It is the ultimate source of heat and energy used by mankind.
7. Produces the antirachitic vitamin A in the green leaf.

The last is akin to the artificial production of the vitamin by exposure of certain substances to ultra-violet light. They include cholesterol, milk, green vegetables and other foodstuffs. Indeed, rickets can be cured by ultra-violet light.

*Proteins.* The nitrogen supply to plants is chiefly due to bacteria. Using the sugar in leguminous plants, they are able to fix nitrogen derived from the soil and air. The nodules on the finer roots are composed of bacteria which are subsequently digested by the plant when using up the nitrogen fixed by the bacteria. Clovers, peas and beans are therefore employed for rotation of the crops. Some bacteria produce nitrites which others convert into nitrates, the chief source of nitrogen for green plants.

The continuity of life depends on sunlight, chlorophyll, and bacteria. Reproduction in plants is of two kinds: vegetative, as in the yeast cell, in which there is continuity without change; and sexual, in which variation, change and progress are possible by the interaction of sex. The sexual reproduction of plants is the prototype of the like dominant process in animals, and in plants and lower animals we find transitional forms. Cilia are found in the male reproductive cells of seaweeds and mosses, and in a fern the antherozoid swims through the water to the ovum like a spermatozoon. Alternation of generations can be traced in lower plants and in a few animals. The asexual spores under the leaf of a bracken fern drop off and grow into a prothallus which produces both ova and antherozoids. The ova and spermatozoa of jelly-fish produce an asexual generation, the hydroid, before the sexual generation again appears. Incubation occurs in flowering plants, the prothallus being hidden within the parent like an embryo, with a store of starch and protein to draw upon. Most plants and many animals are hermaphrodite. When a certain parasite infects the hermit crab ova appear in the testis and the male assumes a female appearance. Sex may alternate. The oyster appears to be at first male, then female, and then male again. Parthenogenesis is common in plants. In some creatures males are unknown, in others (insects and lower crustaceans) there may be many parthenogenic generations before a male appears. In bees the queen and workers are products of fertilization, the males or drones are born parthenogenetically. Parthenogenesis may be induced artificially by feeding (paramœcium) or by employing extract of brain or pancreas. Certain unfertilized eggs can be made to develop to the larval stage by altering the saline constituents of the surrounding medium; tadpoles can be produced by pricking frogs' eggs with a needle dipped in salt; and the eggs of sea-urchins will give twins on altering the salts in the water.

Diesase is an abnormal change in the natural continuity of life, and our profession is concerned with a knowledge of what is normal and what is morbid. Human anatomy and physiology are largely comparative, and in the study of diseases, especially tropical ones, the life processes of other creatures have to be considered. Organisms like the amœba are examples of early specialization of function. Motility and prehension are provided by pseudopodia; digestion is indicated by particles of food within the protoplasm; there is a contractile vesicle which acts as an excretory organ; and reproduction takes place by nuclear and cell division.

In the specialization of tissues we find continuity. Muscle and nerve are directly continuous in the embryo with little apparent differentiation between them. In the human adult the sympathetic nerve fibres of the sino-auricular node of the heart, and the plexuses of Meissner and Auerbach in the intestine are intermediate between muscle and nerve in appearance and physiological action; it is impossible to say exactly where one begins and the other ends. In invertebrates some muscles are for action and others for tone or posture. The researches of the late Dr. J. I. Hunter and others have shown that the dual type of innervation exists in vertebrates also, the skeletal muscles containing contractile fibres innervated by somatic nerves and fibres for maintaining tone or posture supplied by sympathetic nerves. Division of sympathetic rami communicantes has been proposed and carried out for selected cases of spastic rigidity; and in medicine "tone" has now to be distinguished as of two kinds, contractile and postural, ramisection being suitable for the latter only.

The relationship between the sympathetic system and the adrenals was summarised by the late Dr. W. H. Gaskell in the following words: "The sympathetic system arose from nerve cells containing adrenaline . . . when these cells left the central nervous system to become peripheral, they left not as single cells but as two separate cells, one of which contained all the adrenaline and formed the chromaffine system, and the other the nerve cells of the sympathetic system of the vertebrate." In a personal communication Dr. Gaskell wrote that he regarded the vertebrate brain as representing the liver of invertebrates. This takes us a long way back for the source of the cholæmic temperament of some individuals.

It is a remarkable fact, and an evidence of continuity, that when diabetes is produced by certain pancreatic deficiency, the need of the individual can be made good by Insulin derived from fish, just as myxœdema may be prevented by giving sheep's thyroid. Equally striking is the fact that the pituitary is concerned with growth in animals, and its extract increases the reproduction of the ciliated slipper animalcule (paramœcia).

The study of embryology explains the origin of certain diseases, particularly those associated with the mandibular slit and branchial clefts, dermoids, spina bifida and the like.

The opening of a meningocele, the sinus rhomboidalis of a fowl and the blastopore of an inverted unicellular organism are homologous. In the broad ligament of the female there exists the vestigial duct of Gartner, the lower part of which may give rise to cysts, particularly in oxen. J. H. Goodall has investigated this structure in cows, sheep, pigs, etc., and after examining some thousands of microscopic sections he concludes that the duct of Gartner is morphologically continuous with the tubules forming the ova. It would seem that the provision of a uterine incubation chamber in placentalia entailed the passage of ova by another route, viz. through the pelvic peritoneum to the Fallopian tube, although ova might have passed down the duct of Gartner just as the sperm passes down the vas deferens, in which case vertebrates in general could have continued to lay eggs and suckle their young, as is still done by a few.

The changes in life make for variation and progress. The individual has to die, that new and better life may be born. The advantage is often reciprocal. The embryo, forming the decidual cells from its chorion, burrows into the maternal tissues and contributes a large share to the formation of the placenta; and the offspring of Life gives birth to maternal affection in the parent, and in its day and in due time learns the true meaning of faith and hope and love.

So, in the life of a society, new and young blood gives fresh life to the body corporate, and we progress in the light of fellowship and the changes brought about by intellectual union.

